# Pharmacogenomic Markers of Methotrexate Response in the Consolidation Phase of Pediatric Acute Lymphoblastic Leukemia Treatment

**DOI:** 10.3390/genes11040468

**Published:** 2020-04-24

**Authors:** Nikola Kotur, Jelena Lazic, Bojan Ristivojevic, Biljana Stankovic, Vladimir Gasic, Lidija Dokmanovic, Nada Krstovski, Goran Milosevic, Dragana Janic, Branka Zukic, Sonja Pavlovic

**Affiliations:** 1 Laboratory for Molecular Biomedicine, Institute of Molecular Genetics and Genetic Engineering, University of Belgrade, Vojvode Stepe 444a, 11 000 Belgrade, Serbia; nikola0104@gmail.com (N.K.); bojan.ristivojevic7@gmail.com (B.R.); bi.stankovic@gmail.com (B.S.); vlada.gasic42@gmail.com (V.G.); branka.petrucev@gmail.com (B.Z.); 2 Department of Hematology and Oncology, University Children’s Hospital, University of Belgrade, Tirsova 10, 11 000 Belgrade, Serbia; lazic.jelena@gmail.com (J.L.); lidija.dokmanovic@udk.bg.ac.rs (L.D.); nada.krstovski@udk.bg.ac.rs (N.K.); dr.gmilosevic@gmail.com (G.M.); dragana.janic@udk.bg.ac.rs (D.J.); 3 Medical Faculty, University of Belgrade, Doktora Subotica 8, 11 000 Belgrade, Serbia

**Keywords:** pharmacogenomics, pediatric ALL, methotrexate, pharmacokinetics, drug-related toxicity

## Abstract

Methotrexate (MTX) is one of the staples of pediatric acute lymphoblastic leukemia (ALL) treatment. MTX targets the folate metabolic pathway (FMP). Abnormal function of the enzymes in FMP, due to genetic aberrations, leads to adverse drug reactions. The aim of this study was to investigate variants in pharmacogenes involved in FMP and their association with MTX pharmacokinetics (MTX elimination profile) and toxicity in the consolidation therapy phase of pediatric ALL patients. Eleven variants in the thymidylate synthetase (*TYMS*), methylenetetrahydrofolate reductase (*MTHFR*)*,* dihydrofolate reductase (*DHFR*), *SLC19A1* and *SLCO1B* genes were analyzed in 148 patients, using PCR- and sequencing-based methodology. For the Serbian and European control groups, data on allele frequency distribution were extracted from in-house and public databases. Our results show that the A allele of *SLC19A1* c.80 variant contributes to slow MTX elimination. Additionally, the AA genotype of the same variant is a predictor of MTX-related hepatotoxicity. Patients homozygous for *TYMS* 6bp deletion were more likely to experience gastrointestinal toxicity. No allele frequency dissimilarity was found for the analyzed variants between Serbian and European populations. Statistical modelling did not show a joint effect of analyzed variants. Our results indicate that *SLC19A1* c.80 variant and *TYMS* 6bp deletion are the most promising pharmacogenomic markers of MTX response in pediatric ALL patients.

## 1. Introduction

Acute lymphoblastic leukemia (ALL) is a disease characterized by abnormal immature B or T lymphocytes—lymphoblasts. ALL accounts for 25% of childhood cancers. Childhood ALL is treated according to standardized protocols, such as the Berlin-Frankfurt-Munster protocol (BFM) [1]. Despite the existence of efficient treatment protocols for childhood ALL, 75% of patients experience therapy related adverse effects, and 1–3% of patients die from toxicities of therapy [2].

Methotrexate (MTX) is one of the staples of standardized protocols of pediatric ALL treatment. MTX is used in every phase of the BFM protocol: in the remission induction (intrathecal administration), the consolidation and the maintenance therapy phase [3]. In the consolidation phase, patients receive high dose of MTX and after each dose, MTX pharmacokinetics is evaluated [3]. High plasma MTX concentration is associated with MTX therapy related toxicities. It is not expected that improvement of disease outcome and reduction of adverse effects and toxicities will be achieved by eliminating MTX from therapy protocols, but by finding a way to calibrate the dosage of MTX.

**Figure 1:**
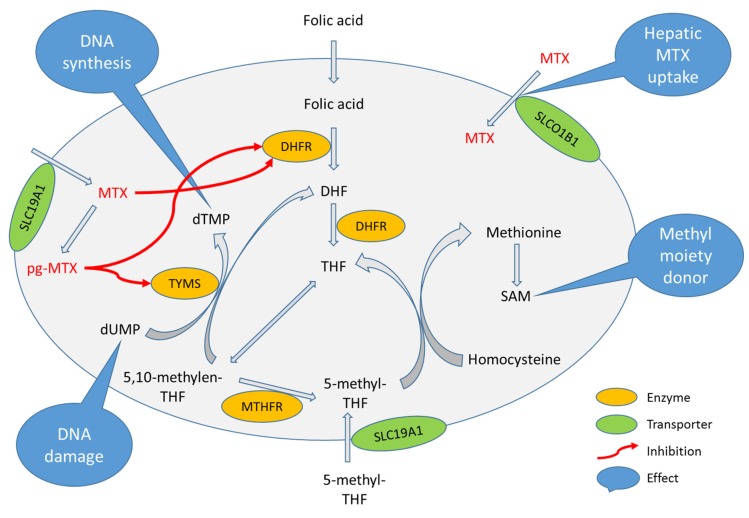
Folate metabolic pathway disrupted by methotrexate (MTX). MTX—methotrexate; pg-MTX—polyglutamated MTX; THF—etrahydrofolate; DHF—dihydrofolate; SAM—S-adenosyl methionine; DHFR—dihydrofolate reductase; TYMS—thymidylate synthetase; MTHFR—methylenetetrahydrofolate reductase; SLC19A1—Solute Carrier Family 19 Member 1; SLCO1B1—Solute Carrier Organic Anion Transporter Family Member 1B1.

MTX is a folate antagonist, and its main therapeutic effect is an inhibition of DNA synthesis. Folate metabolic pathway (FMP) is affected by MTX in a well-defined way (Figure 1). MTX and its polyglutamate forms inhibit key enzymes in folate metabolism, dihydrofolate reductase (DHFR) and thymidylate synthetase (TYMS), by the mechanism of competitive inhibition [4]. The DHFR enzyme replenishes the reserves of the reduced folate form by converting the folic acid and dihydrofolate into tetrahydrofolate (THF). There are several bioactive forms of reduced folate, including 5,10-methylen-THF and 5-methyl-THF, used for thymidylate synthesis (catalyzed by TYMS) and methionine synthesis, for which methylenetetrahydrofolate reductase (MTHFR) enzyme is needed. Methionine is a precursor of S-adenosyl methionine (SAM), which is used for the reactions of methylation, including the methylation of DNA. A lack of THF related to MTX therapy inhibits the numerous processes of DNA synthesis and methylation, thus leading to cell death (therapeutic effect of MTX). Moreover, pharmacokinetics of MTX and other folates is of the essence when it comes to dosing, in order to prevent adverse drug reactions (ADR) [5]. Membrane transporters of solute carrier (SLC) superfamily are important for MTX distribution and clearance. SLC19A1 transporter is responsible for MTX entering the cell, while SLCO1B1 transporter is involved in MTX elimination via bile [6].

Abnormal function of any of the enzymes and transporters in the FMP, due to genetic aberrations, can influence MTX drug efficacy and toxicity and lead to ADR [7]. Pharmacogenomics (PGx) investigates an influence of the individual genetic signature on the response of a patient to treatment [8]. Therefore, pharmacogenomics of MTX needs to be studied in order to determine if it can be implemented in treatment protocols, with the aim to administer the right dose to each pediatric ALL, based on the variants present in the pharmacogenes [9].

The most extensively studied pharmacogenes associated with patients’ response to MTX are *DHFR*, *TYMS*, *MTHFR*, *SLC19A1* and *SLCO1B1*. Numerous variants in these pharmacogenes have been found to be associated with different prognosis and probability of toxic events after MTX administration. Several studies found polymorphisms in *DHFR*, *MTHFR*, *TYMS*, *SLC19A1* and *SLCO1B1* to be significantly associated with event-free survival (EFS) of ALL patients [10,11,12,13,14]. The toxic events can be gastrointestinal, hepatic, neurological, hematologic, etc. There have been numerous studies in favor of classifying some of variants as biomarkers of MTX induced toxicity [12,13,15,16,17,18,19].

Several studies investigated MTX plasma concentration as a surrogate marker of MTX toxicities in association with variants in MTX pharmacogenes, without taking into account clinical signs of MTX related toxicity [16,18]. Other studies have dealt with laboratory and clinical features of patients to establish severity of MTX toxicity, the majority of which included limited number of ALL patients. Meta-analyses were carried out in order to address limited number of patients in those studies. Meta-analyses which included variants of *MTHFR*, *TYMS* and *SLC19A1* pharmacogenes [20,21,22,23] could not definitely disregard or establish analyzed variants as MTX pharmacogenomic markers of ALL treatment.

In order to tackle these issues, we aimed to investigated genetic variants in pharmacogenes involved in the FMP, *DHFR, TYMS, MTHFR, SLC19A1* and *SLCO1B1*, and their association with MTX pharmacokinetics and toxicity in the consolidation phase of treatment of pediatric ALL patients in the context of the Serbian population, making this study the first of its kind.

## 2. Materials and Methods

### 2.1. Subjects

This study enrolled childhood ALL patients diagnosed between 2003 and 2012 at University children’s hospital, University of Belgrade, Serbia. Patients were treated according to the ALL IC-BFM 2002 or the ALL IC-BFM 2009 protocol (Ethic Code Number 017/6-990/67). Only patients who reached consolidation phase were included in the study. The study was approved by the University children’s hospital Ethic Committee, and was carried out in accordance with the Declaration of Helsinki. The control group comprised of 238 individuals of Serbian origin. Informed consent was obtained from the parents or guardians of each patient, as well as from the control group subjects.

### 2.2. Pharmacokinetics of MTX in the Consolidation Therapy Phase

In the consolidation phase, patients receive 4 doses of MTX, either 2 g/m^2^—medium dose MTX (MD-MTX) or 5 g/m^2^—high dose MTX (HD-MTX) [3]. High risk (HR) patients received HD-MTX, while the others received either MD-MTX (mM protocol) or HD-MTX (M protocol), depending on immunophenotype, risk class and randomization. The estimated risk of each patient (standard, intermediate or high risk) was assessed according to age, white blood cell count, early treatment response and genetic aberrations profile, following BFM protocol guidelines.

MTX plasma concentration was measured only in patients who received HD-MTX, and this data is used to discriminate between normal and slow elimination MTX profile, the latter associated with higher rate of ADR. MTX plasma level that exceed either 1 or 0.4 μmol/L after 36h and 48h, respectively, is indicative of slow MTX elimination, according to the BFM protocol.

### 2.3. MTX Toxicity in the Consolidation Therapy Phase

Multiple organ toxicity has been evaluated after each MD- and HD-MTX dose, which included: oral mucositis (OM) and gastrointestinal tract (GIT), liver, renal, skin and neurological toxicities, grading from 0 (no toxicity) to 4 (severe toxicity). Toxicity was estimated through clinical examination, laboratory and radiological findings according to Common Toxicity Criteria of the National Cancer Institute (Common Terminology Criteria for Adverse Events, version 5.0), as presented in Table S1.

### 2.4. Genetic Variants Detection

In this study, variants in *TYMS*, *MTHFR, DHFR*, *SLC19A1* and *SLCO1B* were analyzed (Table 1). Variants in *TYMS*, *MTHFR, DHFR* and *SLC19A1* were detected, as previously described [11]. The region of the *SLCO1B1* gene containing the rs4149056 variant was amplified in a total reaction volume of 30 μL. The reaction mix contained 10 pmol of each primer (forward: CAGCCATGAGGAACTATGAGTCC; reverse: CAGAGATCCCAGGGTAAAGCC), 20–100 ng of genomic DNA, 0.5 mM of each dNTP (Fermentas), 1 × PCR reaction buffer, 2.75 mM MgCl_2_ and 1 U DNA polymerase (FastGene^®^ Taq DNA Polymerase, NIPPON Genetics EUROPE GmbH, Dueren, Germany). The temperature profile of the PCR reactions was starting with the initial activation of DNA polymerase at 95 °C for 5 min, followed by 35 cycles of 30 s denaturation at 95 °C, 30 s annealing at 57 °C and 30 s elongation at 72 °C, ending with a final extension period of 10 min at 72 °C. The PCR fragments were visualized on 2.0% agarose gel, and subsequently analyzed on an ABI PRISM^®^ 3130 DNA analyzer (Applied Biosystems, Foster City, CA, USA).

Variant detection for the control group of Serbian origin was carried out, as described above, on 105 subjects. For 133 control group individuals, their clinical exomes were sequenced employing TruSight One Sequencing Panel on Miseq platform, and the relevant information was extracted using VariantStudio software (version 3, Illumina, San Diego, CA, USA).

For the European control group, data were extracted from 1000 genome project [24] via Ensembl database, which contains the genome data of 503 individuals of European descent. From the Ensembl database, variant frequencies were extracted of all genes of interest, except *TYMS*. Frequencies of *TYMS* variants in the European population were estimated from British, American, Italian and Portuguese Caucasians [25,26,27,28,29].

### 2.5. Statistical Analysis

All detected variants were tested for the Hardy–Weinberg equilibrium using an exact test. Haplotype phases were determined using maximum likelihood algorithm in Arlequin software (version 3.5.1.3, University of Bern, Bern, Switzerland). Differences in allele frequencies between the Serbian and European control groups were tested using chi square test. Genetic variants in transporter genes (*SLC19A1* and *SLCO1B1*) were tested for association with number of low MTX clearance episodes using liner regression. The association of variants in all analyzed genes were tested for association with toxicity using logistic regression. All probabilities were adjusted for demographic and clinical characteristics, as well as MTX dose. Probability values less than 0.05 were considered statistically significant. Multiple comparison correction of all probabilities was applied using false discovery rate (FDR) method. Association analyses were performed using SPSS (version 21, IBM, Armonk, NY, USA) and R (version 3.4.3, R Foundation for Statistical Computing, Vienna, Austria) software.

## 3. Results

### 3.1. Population PGx

A total of 9 single nucleotide variants were analyzed in the promoter of *DHFR* and the coding region of *MTHFR*, *SLC19A1* and *SLCO1B1* genes. One variable number of tandem repeats (VNTR) and one indel, both in UTRs of *TYMS* gene, were also included in analyses. Variant frequencies of childhood ALL patients, as well as control groups of Serbian and European origin, are presented in Table 1. The Hardy–Weinberg equilibrium testing showed that the *TYMS* VNTR variant (rs34743033) in the ALL group and *MTHFR* c.677C>T in the control group were not in the equilibrium. However, after FDR correction for multiple testing, none of the analyzed variants showed statistically significant violation of Hardy–Weinberg equilibrium. Next, we tested for differences in allele frequency distribution between the Serbian and European control groups, and we did not find any statistically significant dissimilarity (Table 1).

### 3.2. Patients

The childhood ALL cohort comprised of 148 patients age 0.9–17.6 years, median 5.5 years, with 94 boys (60.8%). One hundred and eleven patients (75.0%) were treated according to the ALL IC-BFM 2002 protocol, and the others were treated in accordance with the current ALL IC-BFM 2009 protocol. MTX treatment during the consolidation phase did not differ between these two protocols. There were 32 standard risk, 96 intermediate risk and 20 high risk patients. In consolidation, 102, 28 and 18 patients were treated according to the mM, M and HR protocol, respectively. Two patients classified in HR group were treated according to mM and M protocol, because ADR were reported in the previous phase of the therapy.

### 3.3. MTX Kinetics during Consolidation Therapy Phase

**Figure 2:**
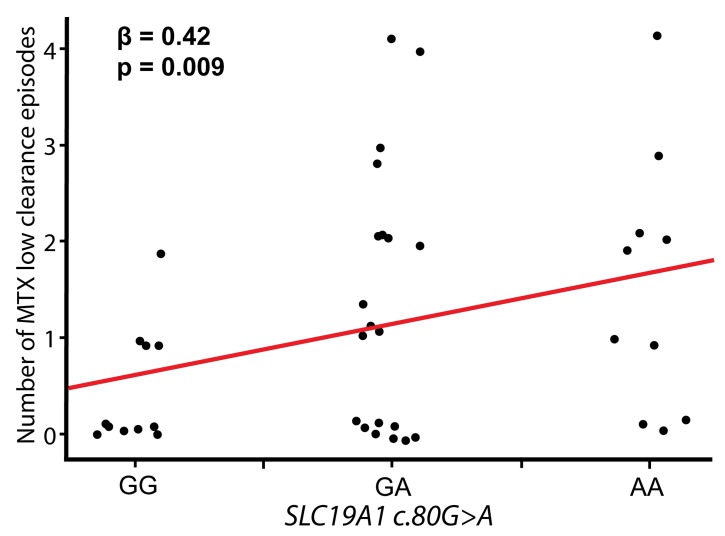
Number of MTX low clearance episodes in *SLC19A1* c.80G>A genotype carriers.

MTX plasma level data in the consolidation phase was available for 41 of 46 patients, who received HD-MTX protocol (28 according to M and 18 according to HR protocol). MTX plasma concentration 36 h and 48 h after each of four HD-MTX doses was used to determine MTX elimination profile (normal or low). Data were available to evaluate MTX elimination profile after 128 HD MTX doses (on average 3.2 per patient). For each patient, MTX elimination profile after each HD-MTX was used to determine number of MTX low clearance episodes (from 0 to 4). In patients for whom MTX elimination profile was not complete after each of the four HD-MTX doses, missing data were estimated taking into account available data for that patient. (For instance, if two normal and one low MTX elimination profile was noted, the number of MTX low clearance episodes would be estimated to be 1/3*4 = 1.33). The number of MTX low clearance episodes was correlated against variants in transporter genes (*SLC19A1* and *SLCO1B1*). Our results show that with each A allele of *SLC19A1* c.80 variant, the odds for having one additional slow MTX elimination episode increased by around 40% (linear regression, *p* = 0.009, β = 0.42 adjusted for age, gender and protocol in consolidation) (Figure 2). The result remained statistically significant after FDR correction. The *SLCO1B1* c.521 variant was not associated with MTX elimination (*p* = 0.85). In order to examine joint effect of *SLC19A1* and *SLCO1B1* variants in MTX elimination, we included both variants in the liner regression model. Results showed that *SLC19A1* remained significant predictor of the MTX elimination profile (*p* = 0.014, β = 0.42), while *SLCO1B1* variant did not contribute to the model (*p* = 0.94, β = 0.012).

### 3.4. MTX Toxicity during Consolidation Therapy Phase

After each MD or HD-MTX dose, toxicity was evaluated according to the Common Toxicity Criteria of the National Cancer Institute criteria. HR patients were excluded from toxicity analyses, because they receive aggressive therapy comprising of multiple chemotherapeutics, thus, attributing observed toxicity to MTX would be challenging. Therefore, for toxicity evaluation, only patients who were treated according to mM and M protocol are included (*n* = 130). For each non-HR patient, after each MD and HD-MTX dose, toxicity of multiple organs was evaluated, and data regarding the highest toxicity grade per organ is presented in Table 2.

Next, we evaluated genetic markers in the folate pathway as predictive markers of toxicity attributed to MD-MTX and HD-MTX therapy. Patients were divided into low (grade 0–1) and high toxicity groups (grade 2–4), and genetic markers were tested for association using a recessive genetic model for variants in *TYMS*, *MTHFR*, *SLC19A1* and *SLCO1B1* genes. Five *DHFR* variants located less than 400 bp apart and in strong mutual linkage disequilibrium were analyzed as haplotypes. To evaluate the association of a haplotype with a certain type of toxicity, patients with the haplotype of interest were compared to other patients who did not carry that haplotype. The association analyses with genetic markers were carried out for oral mucositis, hepatotoxicity and GIT toxicity, because a considerable number of patients were affected with these kinds of ADR (Table 2). The results showed that non-HR patients with an AA genotype of *SLC19A1* c.80G>A variant were almost 10 times more likely to experience grade 2 or higher hepatotoxicity than the non-HR patients with GG or GA genotype (*p* = 0.011, logistic regression, adjusted for age, gender and MTX dose). Non-HR patients homozygous for *TYMS* 6bp deletion were more than four times more likely to experience grade 2 or higher GIT toxicity than the non-HR patients with other genotypes (*p* = 0.020, logistic regression, adjusted for age, gender and MTX dose). Other associations were not statistically significant (Table 3 and Table S2). When probabilities were corrected for multiple comparisons using FDR, no association remained statistically significant.

Next, we analyzed selected genetic variants in the multivariate logistic models with an aim to examine their joint effect on MTX toxicity. We did not find any significant contribution of variants when they were analyzed together (data not shown), other than that already identified in the previous analysis (Table 3). We concluded that these variants do not interact in prediction of MTX toxicity.

## 4. Discussion

Research efforts in pediatric ALL are focused not only towards etiopathology of the disease and new therapeutic options comprehension, but also towards finding ways to avoid adverse side effects of therapy, therapy toxicity, in a way to efficiently predict them [30]. Our study has contributed by validation of genetic variants, candidates for PGx markers associated with MTX pharmacokinetics and toxicity in pediatric ALL patients in Serbia. Our study validated the *SLC19A1* c.80 variant as being associated with delayed methotrexate clearance and the *TYMS* 6bp deletion variant as a risk of developing gastrointestinal toxicity in pediatric ALL patients.

The *SLC19A1* gene is located on chromosome 21q22.3, and codes for the SLC19A1 transporter [31]. The main route of cellular folate intake is by active transport using folate transporters, including SLC19A1. The most represented folate form in circulation, 5-methyl-THF, as well as MTX, enter the cell efficiently via SLC19A1 [31,32]. *SLC19A1* c.80 G>A is the most common and the most studied *SLC19A1* variant [33]. This variant is located in exon 2 of the *SLC19A1* gene, and affects amino acid change p.His27Arg. As the variant position is in the region coding for transmembrane domain of the SLC19A1 protein, it could disrupt the structure itself, influencing the kinetics of the SLC19A1 substrate. Several variants identified in the region coding for the transmembrane domain of SLC19A1 may influence the capacity of SLC19A1 to transport MTX, preventing the transporter from situating itself in a proper position in the cell membrane [34,35,36,37,38,39,40]. An effect of the *SLC19A1* c.80 variant to reduce the SLC19A1 MTX transport was investigated in transport-impaired K562 cells transfected with both His27 and Arg27 transporters [41]. Two-fold differences were detected between the Arg27 and His27 transporters in the interaction affinity of different anti-folates. The results suggested that there were at most minor functional differences between Arg27 and His27 transporters in terms of substrate affinities and transport efficiencies. *SLC19A1* c.80 G>A variant reduces the SLC19A1 transfer mediated efficacy of both natural folates and MTX, however, the functional significance and mechanism have to be yet fully elucidated [30,42].

Because of the very important physiologic function of the SLC19A1 transporter, its reduced activity could influence the onset of certain diseases associated with low intracellular folate intake such as malignant, cardiovascular and neurological diseases, as well as modulate the efficacy and toxicity of MTX. Studies examining the association between the genetic variant SLC19A1 c.80A in children with ALL and disease outcome have yielded conflicting results. *SLC19A1* c.80 AA and GA genotypes are associated with ALL relapse and poor survival [43]. One study reported that of all the genetic variations associated with the folate pathway, the presence of the *SLC19A1* c.80AA genotype conveys the largest risk for developing ALL [44], while another study showed the opposite results: a protective effect of *SLC19A1* c.80 AA genotype [45]. A study found an association between *SLC19A1* c.80 AA genotype and a higher probability of staying in remission, compared with GG or GA variants, and a lower likelihood of relapse among 500 children with ALL, as well as its effect on greater bone marrow toxicity [46]. Furthermore, the relationship between a greater number of copies of the gene present in ALL patients with trisomy 21 or hyperdiploid ALL, and a better outcome or greater MTX toxicity, was shown in the same study, possibly due to greater intracellular polyglutamated MTX in this setting.

Our results in pediatric ALL patients receiving HD-MTX doses have shown that the possibility for having one additional low clearance episode increases by 40% with each A allele of *SLC19A1.c80* variant. This finding suggests that *SLC19A1.c80* variant is a strong candidate for MTX kinetics PGx marker.

Additionally, our results demonstrated that the SLC19A1 c.80G>A variant could be a PGx marker for hepatotoxicity in pediatric ALL patients. According to a meta-analysis, it was demonstrated that G80A, a variant which affects the activity of SLC19A1, is not a good marker of MTX-related toxicity in pediatric ALL [33]. Another study concluded that the same variant is associated with increased MTX toxicity [47]. This is another example of the inconsistency of the assessment of relevance of PGx markers for MTX toxicity.

We have also investigated the association of MTX kinetics during the consolidation with variants in *SLCO1B1*, another transporter encoding gene. MTX clearance was associated with numerous variants in *SLCO1B1* [16]. The functional variant c.521 in *SLCO1B1* gene has been suggested to play an important role in methotrexate clearance and related toxicities in patients with pediatric ALL in several studies [15,17,18,48]. However, some studies did not replicate those results [49,50,51].

Our results, pointing out that the *SLCO1B1* c.521 variant was not associated with MTX elimination, do not support the proposed role of the c.521 variant in decreased MTX clearance and the development of related toxicities. We hypothesize that the lack of consistency across the studies could be due to the fact that the pharmacokinetics of methotrexate can be influenced by both non-genetic and genetic factors, since it has been shown that all *SLCO1B1* variants account for 10.7% of the population variability in MTX clearance [17]. Among the non-genetic influences, the differences in treatment protocols, estimations of methotrexate toxicity and homogeneity of the studied populations could be the main sources of differences across the studies.

*TYMS* encodes the enzyme essential for DNA replication and repair, converting dUMP (uridine monophosphate) into dTMP (thymidine monophosphate). As the function of TYMS enzyme in fast-dividing cells is of crucial importance, the TYMS enzyme is the target in the treatment of various types of malignant and inflammatory diseases. TYMS enzyme inhibitors include pyrimidine analogues, fluoropyrimidines and folate analogs, including MTX [52,53]. The most studied genetic variations in the *TYMS* gene are located in the 5′UTR and 3′UTR regions, and influence mRNA stability, localization and translational efficiency [54]. A minisatellite region, usually containing three or two repeats, each 28 base pairs (bp) long, is located in the 5′UTR region of the *TYMS* gene. The allele with two repeats (2R) has been shown to cause decreased translation of TYMS relative to the three-repeat (3R) allele [55], but the exact mechanism is not fully known. It has been reported that patients with rheumatoid arthritis and *TYMS* 3R3R genotype show a weaker response to MTX therapy [56]. The 3′UTR region of the *TYMS* gene contains and 6bp-long indel. Alleles with deletion exhibit decreased mRNA stability and result in lower levels of thymidylate synthetase, due to higher degradation susceptibility [57]. Polyglutamate forms of MTX bind directly to the TYMS enzyme and thus inhibit it [58]. This mechanism is related to the observed finding of increased gastrointestinal toxicity. Intestinal mucositis, bleeding and peptic ulcers are well-known gastrointestinal adverse effects of MTX. The pathogenic mechanism underlying this mucositis remains unknown, but immune response and increased blood concentration could be considered as the causes [59]. Polyglutamate forms of MTX bind directly to the TYMS enzyme, and thus inhibit it. If the cell is naturally enriched in the enzyme, due to the presence of the TYMS allele causing increased gene expression, less toxicity but also less effectiveness of therapy is expected [60]. Conversely, patients harboring the TYMS gene variants causing decreased gene expression, display a higher likelihood of adverse drug reactions [61]. We believe that our finding, increased gastrointestinal toxicity in ALL patient carriers of TYMS 6bp deletion, is a result of the interaction of MTX and TYMS, whose low expression level enables more effective action of MTX, leading both to a better therapeutic effect and more prominent toxicity.

Our results have demonstrated that among children with ALL, homozygous carriers *TYMS* 6bp deletion, were more than four times more likely to experience grade 2 or higher GIT toxicity than the non-HR patients with other genotypes. In addition, non-HR patients with the AA genotype of the *SLC19A1* c.80G>A variant were almost 10 times more likely to experience grade 2 or higher hepatotoxicity than the non-HR patients with GG or GA genotype. It was already shown that *SLC19A1* c.80A allele was associated with gastrointestinal MTX toxicity in children with ALL [62]. Our findings are in line with the published literature, suggesting that the *TYMS* and SLC19A1 variants could be explored as pharmacogenetics variants.

We have tried to highlight our results in population-specific genetic background. It has been previously shown that the allele frequencies of several pharmacogenes are significantly different when the Serbian population is compared with other European populations [63]. However, for the variants in pharmacogenes analyzed in this study, no statistically significant dissimilarity was found. Therefore, the population-specific genetic background of the patients included in this study did not contribute to particular findings reported here. The fact that pharmacogenetics variants mostly are not cause of the disease is furthermore seen from the variant frequencies around 0.5 for TYMS rs34743033 and SLC19A1 rs1051266. The majority of variants analyzed in our study had a variant frequency of around 30% (TYMS rs34489327, MTHFR rs1801133 and rs1801131, DHFR rs442767, rs1643641, rs1650695 and rs1650696). These relatively high numbers could indicate the importance of the relevant pharmacogenetics markers in European populations, including the Serbian population.

Some limitations should be considered when interpreting the results of this study. They include relatively small number of pediatric ALL patients—only 130 for toxicity study and 41 for methotrexate clearance follow-up. The candidate gene approach we used could not reveal new potential pharmacogenes but only assess already proposed ones. The study did not directly assess gene expression nor protein levels in the FMP, as these pharmacotranscritomics markers are recognized as important factors in methotrexate clearance and toxicity [11,57,64]. However, our findings contribute to the knowledge of PGx of MTX. Most previous studies have analyzed the association of particular genetic variants in FMP with MTX plasma levels or MTX resistance. The findings of our study that the *SLC19A1c*.80 variant had significant role in methotrexate clearance and that *TYMS* 6bp variant could be considered a valid pharmacogenetics marker of MTX gastrointestinal toxicity, which represents an important piece in a complex MTX PGx puzzle.

MTX toxicity has been recognized as potentially life-threatening event during chemotherapy for a while. International guides that identify and grade MTX toxicity [65], already integrated into modern treatment protocols for ALL in children [66], still need evidence-based revision and continuous upgrade. It is demonstrated again that the pharmacogenomics of MTX is a complex research field, since the MTX/folate metabolic pathway is not a simple one [7,58,67]. A number of polymorphic genes encode enzymes and transporters involved in different pathway branches of folate metabolism. Moreover, gene–gene interactions in folate-related genes were observed, and could contribute to complex interdependencies between the pathway participants [68]. Additionally, there are gene-nutrient interactions and the evident modifying role of dietary components on the sophisticated folate machinery [69].

It appears that genome wide approach has not been successful in defining valid PGx markers due to limited statistical power. A candidate gene approach is incapable of identifying new genes and genetic variants that could be used as drug targets or predictive markers [70], because they cannot include all the relevant aspects of MTX metabolism. Nevertheless, continuous technology progress provides us with more genomic and clinical data that area likely to help researchers understand and predict adverse reactions to therapy, as well as contributing to better outcomes of the disease. Molecular profiling of patients using high-throughput technology, as well as the implementation of artificial intelligence through advanced bioinformatics tools and predictive machine learning algorithms, would enable us to create reliable genetic panels for routine clinical pharmacogenomics testing, to be used before administering different cytotoxic drugs, combinations of drugs or drugs used in different phases of therapeutic protocols for pediatric ALL patients [2]. It is a long way ahead to reach personalized treatment in everyday clinical practice, but pediatric ALL is one of the most encouraging examples that it is achievable.

## Figures and Tables

**Table 1:** Folate pathway genetic variant frequencies of Serbian acute lymphoblastic leukemia (ALL) patients, the Serbian control group and the European descent control group. For the European control group, data were extracted from 1000 genome project for variants in all genes of interest except thymidylate synthetase (*TYMS*). Frequencies of TYMS variants in European population was estimated from British, American, Italian and Portuguese Caucasians [25,26,27,28,29].

Genetic Variants	Genotype	Serbian Childhood ALL Patients				Serbian Control Group				European Caucasian Population			*p* Value ^b^
		N	%	HW	MAF (%)	N	%	HW	MAF (%)	N	%	MAF (%)	
*TYMS*^a^ rs34743033 28bp VNTR	3R/3R	49	33.1	0.047	45.7	33	31.7	0.81	42.8	981	29.9	46.3	0.39
	3R/2R	61	41.2			51	49.0			1566	47.7		
	2R/2R	37	25.0			19	18.3			735	22.4		
	3R/4R	1	0.7	NA	NA	1	1.0	NA	NA	NA	NA	NA	NA
*TYMS* rs34489327 6bp indel	Ins/Ins	70	47.3	0.57	33.1	47	45.2	0.92	33.2	1532	46.3	32.8	0.92
	Ins/Del	61	41.2			45	43.3			1386	41.8		
	Del/Del	17	11.5			12	11.5			394	11.9		
*MTHFR* rs1801133 c.677 C>T	C/C	70	47.3	0.57	32.1	113	47.5	0.013	33.8	204	40.6	36.5	0.32
	C/T	61	41.2			89	37.4			231	45.9		
	T/T	17	11.5			36	15.1			68	13.5		
*MTHFR* rs1801131 c.1298 A>C	A/A	71	48.6	0.252	31.4	115	48.3	0.761	30.9	239	47.5	31.3	0.86
	A/C	57	39.0			99	41.6			213	42.3		
	C/C	18	12.3			24	10.1			51	10.1		
*DHFR* rs442767 -680 C>A	C/C	63	44.7	1	31.4	45	43.3	0.86	34.6	232	46.1	31.9	0.45
	C/A	63	44.7			46	44.2			221	43.9		
	A/A	15	10.6			13	12.5			50	9.9		
*DHFR* rs1643641 -675 A>G	A/A	74	52.5	0.31	27.4	55	52.9	0.31	26.0	286	56.9	25.2	0.82
	A/G	53	37.6			44	42.3			180	35.8		
	G/G	14	9.9			5	4.8			37	7.4		
*DHFR* rs1650695 -556 T>C	T/T	73	51.8	0.54	27.4	55	52.9	0.52	26.4	285	56.7	25.3	0.74
	T/C	55	39.0			43	41.3			181	36.0		
	C/C	13	9.2			6	5.8			37	7.4		
*DHFR* rs1650696 -464 A>T	A/A	72	51.1	0.68	27.7	55	52.9	0.52	26.4	286	56.9	25.2	0.72
	A/T	56	39.7			43	41.3			180	35.8		
	T/T	13	9.2			6	5.8			37	7.4		
*DHFR* rs408626 -317 A>G	A/A	49	34.8	0.60	28.2	37	35.6	0.99	40.4	150	29.8	45.0	0.22
	A/G	71	50.4			50	48.1			253	50.3		
	G/G	21	14.9			17	16.3			100	19.9		
*SLC19A1* rs1051266 c.80 G>A	G/G	39	26.9	0.40	49.0	72	30.3	0.80	65.7	158	31.4	45.1	0.92
	G/A	67	46.2			116	48.7			236	46.9		
	A/A	39	26.9			50	21.0			109	21.7		
*SLCO1B1* rs4149056 c.521 T>C	T/T	100	71.9	0.06	15.5	100	74.6	1	13.4	351	69.8	16.1	0.28
	T/C	32	23.0			32	23.9			142	28.2		
	C/C	7	5.0			2	1.5			10	2.0		

^a^ Rare 4R allele of *TYMS* variant rs34743033 was disregarded in all analyses. ^b^ Probability obtained after chi square testing for differences in allele frequencies between Serbian and European control groups; HW—Hardy–Weinberg statistics. The number represents *p* value for HW equilibrium testing; NA—not available/not applicable; MAF—minor allele frequency. R—repeats. N—number of subjects

**Table 2:** Number of ALL patients experiencing different grade of toxicity during consolidation phase.

	mM Protocol (MD-MTX), n = 102 Toxicity Grade					M Protocol (HD-MTX), n = 28 Toxicity Grade				
	0	1	2	3	4	0	1	2	3	4
GIT toxicity	61	17	22	2	-	15	3	9	1	-
Oral mucositis	75	19	6	2	-	19	4	5	-	-
Liver toxicity	81	16	4	1	-	21	4	1	1	1
Nephrotoxicity	99	3	-	-	-	23	4	1	-	-
Skin toxicity	102	-	-	-	-	26	-	2	-	-
Neurotoxicity	96	3	3	-	-	26	1	1	-	-

GIT—gastrointestinal tract.

**Table 3:** Genetic variation in folate pathway genes as predictive markers of GIT toxicity, hepatotoxicity and oral mucositis (Toxicity grade 0-1 versus 2-4). To assess the association of genetic variants with medium to severe toxicity, logistic regression was used, and all probabilities were adjusted for age, gender and MTX dose. The recessive genic model was applied for variants in *TYMS*, methylenetetrahydrofolate reductase (*MTHFR*), *SLC19A1* and *SLCO1B1* genes, with the reference group comprising common homozygotes and heterozygotes. Patients with denoted dihydrofolate reductase (*DHFR*) haplotypes were compared against the patients who carried all other haplotypes, the latter group being the reference group for statistical analyses.

Gene	Variant	dbSNP	Alleles	MAF	Oral Mucositis			GIT toxicity			Hepatotoxicity		
					OR	95% CI	*p*	OR	95% CI	*p*	OR	95% CI	*p*
***MTHFR***	c.677 C>T	rs1801133	C/T	0.32	1.54	0.29–8.07	0.61	0.97	0.28–3.41	0.97	NA	NA	1.00
***MTHFR***	c.1298 A>C	rs1801131	A/C	0.32	1.17	0.21–6.52	0.86	0.36	0.07–1.73	0.20	3.37	0.55–20.70	0.19
***TYMS***	28bp repeats	rs34743033	2R–4R	2R: 0.46 4R: 0.003	0.43	0.09–2.13	0.30	0.75	0.29–1.92	0.55	1.57	0.34–7.32	0.56
***TYMS***	6bp indel	rs34489327	Ins/Del	0.32	2.77	0.60–12.73	0.19	4.17	1.25–13.91	0.020	NA	NA	1.00
***DHFR hap1***	promoter region (−680–−317)	rs442767–rs1643641– rs1650695–rs1650696– rs408626	A-A-T-A-G	0.33	0.37	0.09–1.60	0.18	1.02	0.44–2.37	0.96	5.83	0.65–52.61	0.12
***DHFR hap2***			C-A-T-A-A	0.30	8.07	0.97–67.5	0.054	1.42	0.62–3.26	0.41	1.18	0.24–5.65	0.84
***DHFR hap3***			C-A-T-A-G	0.07	0.73	0.08–6.88	0.78	0.65	0.16–2.61	0.55	3.08	0.50–18.90	0.22
***DHFR hap4***			C-G-C-T-A	0.27	1.15	0.30–4.41	0.84	1.19	0.52–2.73	0.68	0.49	0.09–2.70	0.41
***SLC19A1***	c.80 G>A	ra1051266	G/A	0.50	1.09	0.26–4.57	0.90	1.38	0.56–3.40	0.49	9.70	1.70–55.78	0.011
***SLCO1B1***	c.521 T>C	rs4149056	T/C	0.16	NA	NA	1.00	1.65	0.27–10.02	0.59	NA	NA	1.00

GIT—gastrointestinal tract; MAF—minor allele frequency; OR—odds ratio; CI—confidence interval; NA—not applicable because OR could not be estimated.

## Supplementary Materials

The following are available online at https://www.mdpi.com/2073-4425/11/4/468/s1, Table S1: Criteria for grading drug related toxicity according to Common Toxicity Criteria of the National Cancer Institute. Table S2: Childhood ALL patients (n = 130) experiencing low (grade 0–1) or high (grade 2–4) toxicity divided by presence of specific genotype/haplotype and protocol regiment during the consolidation phase of BFM guided therapy.
